# Intranasal infection by SARS-CoV-2 Omicron variants can induce inflammatory brain damage in newly weaned hamsters

**DOI:** 10.1080/22221751.2023.2207678

**Published:** 2023-06-12

**Authors:** Can Li, Wenchen Song, Jasper Fuk-Woo Chan, Yanxia Chen, Feifei Liu, Zhanhong Ye, Alvin Hiu-Chung Lam, Jianpiao Cai, Andrew Chak-Yiu Lee, Bosco Ho-Yin Wong, Hin Chu, David Christopher Lung, Siddharth Sridhar, Honglin Chen, Anna Jin-Xia Zhang, Kwok-Yung Yuen

**Affiliations:** a State Key Laboratory of Emerging Infectious Diseases, Carol Yu Centre for Infection, Department of Microbiology, School of Clinical Medicine, Li Ka Shing Faculty of Medicine, The University of Hong Kong, Pokfulam, Hong Kong Special Administrative Region, People’s Republic of China; b Centre for Virology, Vaccinology and Therapeutics, Hong Kong Science and Technology Park, Shatin, Hong Kong Special Administrative Region, People’s Republic of China; c Department of Infectious Disease and Microbiology, The University of Hong Kong-Shenzhen Hospital, Shenzhen, Guangdong, People’s Republic of China; d Department of Microbiology, Queen Mary Hospital, Pokfulam, Hong Kong Special Administrative Region, People’s Republic of China; e Academician Workstation of Hainan Province, Hainan Medical University – The University of Hong Kong Joint Laboratory of Tropical Infectious Diseases, Haikou, People’s Republic of China; f Department of Pathology, Hong Kong Children’s Hospital, Hong Kong Special Administrative Region, Hongkong, People’s Republic of China; g Department of Pathology, Queen Elizabeth Hospital, Hong Kong Special Administrative Region, Hongkong, People’s Republic of China

**Keywords:** Coronavirus, COVID-19, SARS-coV-2, brain, newly-weaned hamster

## Abstract

Children infected by SARS-CoV-2 Omicron variant may develop neurological complications. To study the pathogenesis in the growing brain, we intranasally challenged newly-weaned or mature hamsters with SARS-CoV-2 Omicron BA.2, BA.5, or Delta variant. Omicron BA.2 and Delta infection produced a significantly lower viral load in the lung tissues of newly-weaned than mature hamsters despite comparable histopathological damages. Newly-weaned hamsters had higher brain viral load, significantly increased cerebrospinal fluid concentration of TNF-α and CXCL10 and inflammatory damages including mild meningitis and parenchymal vascular congestion, despite sparse expression of nucleocapsid antigen in brain cells. Furthermore, 63.6% (28/44) of all SARS-CoV-2 infected newly-weaned hamsters showed microgliosis in olfactory bulb (OB), cerebral cortex, and hippocampus. In infected mature hamsters, microgliosis was observed mainly in OB and olfactory cortex of 35.3% (12/34) of their brains. Neuronal degeneration was found in 75% (33/44) of newly-weaned hamsters affecting multiple regions including OB, olfactory cortex, midbrain cortex, and hippocampus, while such changes were mainly observed in the hippocampus of mature hamsters. Importantly, similar brain histopathology was also observed in Omicron BA.5-infected newly-weaned hamsters. Our study suggested that SARS-CoV-2 may affect the brain at a young age. This kind of brain involvement and histological changes are not virus variant or subvariant specific. Incidentally, a moderate amount of eosinophilic infiltration was observed in the mucosa of nasal turbinate and trachea of newly-weaned hamsters infected by Omicron BA.2 and BA.5 but not Delta variant. This histological finding is consistent with the higher incidence of laryngotracheobronchitis in young children infected by the Omicron variant.

**Summary:**

Intranasal infection of newly-weaned Syrian hamsters by SARS-CoV-2 Omicron variants can lead to brain inflammation and neuron degeneration with detectable low level of viral load and sparse expression of viral nucleoprotein.

## Introduction

The coronavirus disease 2019 (COVID-19) caused by severe acute respiratory syndrome coronavirus 2 (SARS-CoV-2) has affected over 643 million people with about 6.6 million deaths as of 26 December 2022 [1,2]. Over 40 million children under age 14 were infected with 6000 deaths, among which more than 50% were under the age of 5 [3]. Though COVID-19 is primarily a respiratory infection, neurological complications are commonly reported [4]. Up to 80% of COVID-19 patients had neurological symptoms at some point after SARS-CoV-2 infection [5]. The most common neurological manifestations at the onset of the disease are anosmia (loss of smell) due to the destruction of olfactory epithelium and sensory neurons by SARS-CoV-2 [6,7]. Other common neurological symptoms during the disease course include dizziness, headaches, myalgia, hyposmia, impaired consciousness, seizures, and ataxia [8]. Radiological and pathological evidence of brain involvement and the virologic findings in cerebrospinal fluid (CSF) suggested the possibility of direct virus invasion of the central nervous system [9]. Recent studies indicated that nearly one-third of adult COVID-19 patients, even those with mild respiratory symptoms, experienced long-term neurological symptoms which are now considered as long COVID [10,11]. They experienced malaise, debilitating fatigue, and impaired memory or executive function long after their recovery [12,13]. MRI imaging of their brains showed significantly decreased brain size or various degree of brain tissue damage, which are associated with cognitive decline [14]. Moreover, population immunity from natural infection and vaccination was associated with the emergence of immune evasive SARS-CoV-2 variants of which the Omicron variant dominated and replaced other variants by escaping from neutralizing antibodies induced by prototype virus infection or vaccination [15,16]. Since the emergence of Omicron variant, more young children have been infected with mostly mild illness similar to the disease caused by the prototype or previous variants [17,18]. However, among those hospitalized children with COVID-19, 15.0% of them were admitted for neurological complications, this percentage is significantly higher when compared with the 3.8% reported for other SARS-CoV-2 variants during the pre-vaccination period in the United Kingdom [19,20]. One study comparing Delta and Omicron infections in children reported that while Omicron caused relatively milder respiratory manifestation [18], their neurological and psychiatric complications were not reduced [10]. The neurological manifestations reported in children include seizures, encephalitis, and acute necrotizing encephalopathy [20,21]. During the Omicron surge in Hong Kong, neurological manifestations and their severity were significantly increased in children with some fatal outcomes [21]. However, the pathogenesis of Omicron variant in the respiratory and central nervous system in young children is still uncertain. To understand the impact of Omicron infection on the growing brain, we utilized the newly-weaned hamster and studied the histopathological changes after intranasal inoculation of currently circulating variants, including Omicron BA.2, BA.5, and the previous Delta variant, and compared with 8-weeks-old mature hamster. We found that all three SARS-CoV-2 variants caused more frequent inflammatory and degenerative damages in the brain of newly-weaned hamsters than mature hamsters.

## Methods

### Viruses, cell lines, and biosafety

SARS-CoV-2 Omicron BA.2 strain (GISAID accession number: EPI_ISL_9845731), BA.5 strain (GISAID accession number: EPI_ISL_13777658), and Delta strain (GISAID accession number: EPI-ISL-3221329) were isolated from laboratory-confirmed COVID-19 patients in Hong Kong. The viruses were propagated and titrated for plaque forming unit (PFU) in African green monkey kidney cells overexpressing TMPRSS2 protein (Vero E6-TMPRSS2) [22]. All experiments involving live SARS-CoV-2 were conducted in Biosafety Level-3 (BSL-3) laboratory of the University of Hong Kong (HKU) following approved standard operating procedures.

### Animal model

Syrian hamsters, newly-weaned at 3 weeks of age or mature at 8 weeks of age, were obtained from the Centre for Comparative Medicine Research, HKU. All the animal experimental procedures were approved by the Committee on the Use of Live Animals in Teaching and Research of HKU (CULATR #6093-22).

### Intranasal inoculation of virus to hamster

Hamsters under anaesthesia of intraperitoneal injection of ketamine (150 mg/kg) and xylazine (10 mg/kg) were inoculated intranasally with 10^5^ PFU SARS-CoV-2 virus in 50 μl volume per animal as we previously reported [23,24]. Mock-infection controls were given the same volume of phosphate-buffered saline (PBS). At 2, 4, or 7 days post-infection (dpi), the hamsters were euthanized by intraperitoneal injection of pentobarbital sodium (200 mg/kg). CSF, blood, brain, lung, trachea, and nasal turbinate (NT) were harvested for analyses. CSF was collected carefully using 0.3 ml syringe through puncture at the cisterna magna to avoid any blood contamination. Brain was collected after removing calvaria.

### Determination of viral load by real-time RT–PCR (RT-qPCR) and plaque assay

Total RNA was extracted from homogenized brain, lung, and NT. SARS-CoV-2 RNA-dependent RNA polymerase (RdRp) and subgenomic E (Sg E) gene copies were determined by RT-qPCR [25]. The infectious virus in the tissue homogenate was determined by 50% Tissue Culture Infectious Dose (TCID_50_) in Vero E6-TMPRSS2 cells.

### Detection of SARS-CoV-2 spike RNA by RNAScope

*In situ* RNA hybridization to detect virus spike gene in paraffin-embedded brain tissue sections was performed using RNAScope® Probe-V-nCoV2019-S (848561, ACD) according to the manufacturer’s instructions. The sections were examined using the Olympus BX53 microscope.

### Histopathology, immunohistochemistry, immunofluorescence staining of tissue sections, and automated imaging analysis

Formalin-fixed, paraffin-embedded brain, lung, and NT tissues were processed into 4 µm tissue sections and stained with haematoxylin and eosin (H&E) for histopathological examination as previously described [26,27]. Viral antigens and cellular surface markers were detected by immunohistochemistry or immunofluorescence with specific antibodies: home-made rabbit/mouse anti-SARS-CoV-2 nucleocapsid (N) protein antibody [7,28], anti-neuron-specific type β-III tubulin (801202, Biolegend), anti-Ionized calcium-binding adapter molecule 1 (Iba1) antibody (ab178846, abcam). Brain cell DNA fragmentation was labelled using Click-iT® Plus TUNEL assay kit (Invitrogen) according to the manufacturer’s instructions as we described previously [29]. All tissue sections were examined under light or fluorescence microscopy in a blinded fashion. Images were captured with Olympus BX53 fluorescence microscope with DP80 camera and proprietary Cellsens dimension software (Olympus LS, Japan). For quantification of Iba1-positive microglia, immunofluorescence-stained brain sections were examined and 10–20 images were randomly captured in the area of olfactory bulb (OB), cerebral cortex, and hippocampus, respectively. A magnification of 400× image was applied for a correlated resolution of 0.25 μm/pixel with both DAPI and mCherry channels. Exported TIFF format files were analysed with QuPath (Version 0.3.2).

The histological changes in the brain cortex at 4 and 7 dpi after virus infection were examined systematically, recorded, and scored for comparison of the severity using criteria adopted from previous reports with modifications [30]. Briefly, scoring for meningitis: meningeal vessel congestion and increased vascular space cellularity present (1) or absent (0); brain cortex blood vessel congestion, perivascular immune cell infiltration, microgliosis and neuronal degeneration, neuronal necrosis occurred in any anatomical region: absent (0); minimal if found in one anatomical region of the brain section (1); mild if found in more than one region (2); moderate changes occurred in more than one anatomical region (3). There were no cases that can be defined as severe in this study. Lung histological changes were accessed and scored as described previously [28].

### RT-qPCR determination of pro-inflammatory cytokine and chemokine mRNA expressions

Total RNA was extracted from tissues with MiniBEST Universal RNA Extraction Kit (Takara) and reverse transcribed into cDNA with PrimeScript^TM^ RT reagent kit (Takara). RT-qPCR was performed using gene-specific primers (Supplementary Tables 1 and 2) with SYBR Premix Ex Taq II Kit (Takara). The expression of cytokine and chemokine genes was analysed by ΔΔ*C_t_* method, using mock-infected tissue samples as baseline.

### Determination of cytokine in serum and CSF by Elisa

Serum or CSF cytokine and chemokine concentration were determined using hamster IL-6 ELISA kit (MBS700950, Mybiosource), TNF-α ELISA kit (MBS7696475, Mybiosource), and CXCL10/IP-10 ELISA kit (MBS7612365, Mybiosource) according to the manufacturer’s instructions.

### Statistical analysis

All data were analysed with Prism 8.0 (GraphPad Software Inc). Statistical comparison among different groups was performed using Fisher’s exact test, one-way ANOVA, or two-way ANOVA. *p* < 0.05 was considered statistically significant and highlighted in all figures.

## Results

### Omicron BA.2 and Delta replicate at lower levels in newly-weaned hamsters but cause severe respiratory tissue damages

SARS-CoV-2 Omicron variants were reported to have attenuated pathogenesis in respiratory tissues of human and in animal models when compared to previous strains, such as Delta variant [31]. We challenged newly-weaned and mature hamsters with Omicron BA.2 or Delta variant and examined the NT and lung at 2, 4, and 7 dpi. In general, Omicron BA.2 replicated less effectively than Delta variant, which is consistent with previous reports [28].

**Figure 1.**
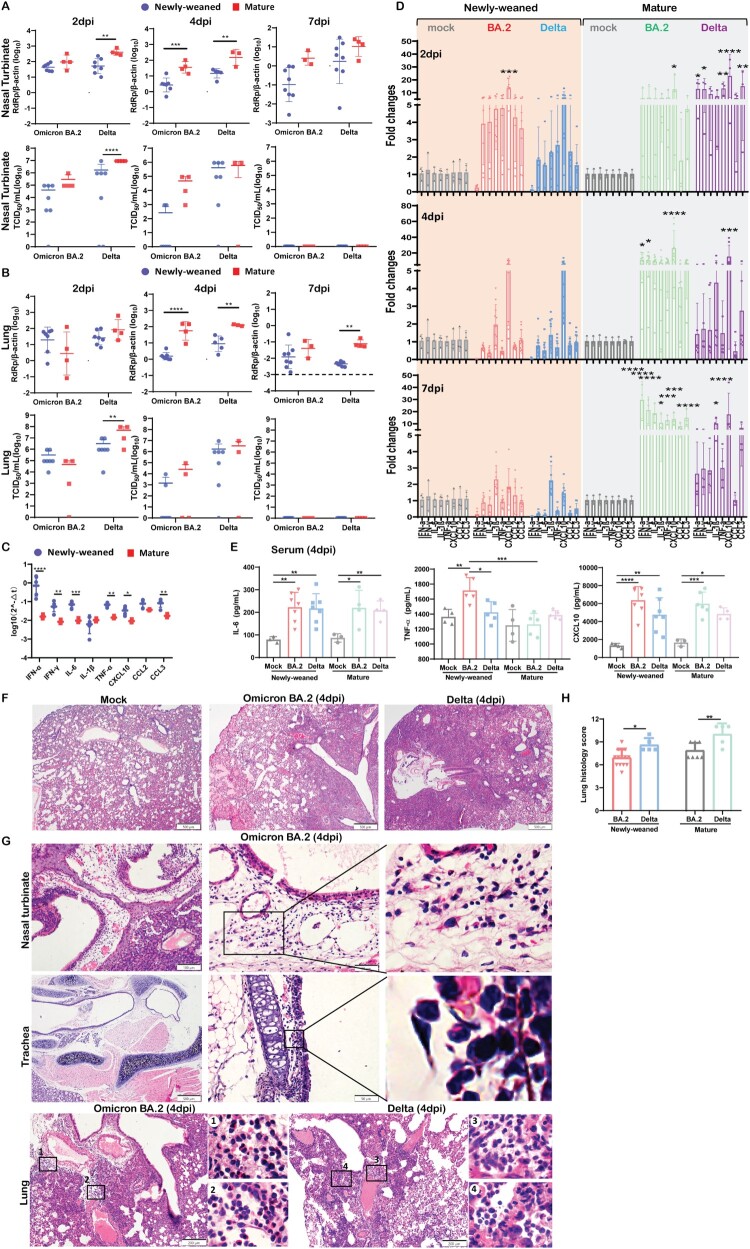
Omicron BA.2 or Delta virus infection in the respiratory tissues of newly-weaned or mature hamsters. (A) Viral genomic RdRp gene copies and infectious virus titre in the NT. *n* = 3–8. (B) Viral genomic RdRp gene copies and infectious virus titre in the lung tissues. *n* = 3–8. Dashed lines are indicated as detection limits. (C) Basal mRNA expression levels of pro-inflammatory cytokine/chemokine in lung of mock newly-weaned or mature hamsters. *n* = 3–5. (D) Relative mRNA expression levels of pro-inflammatory cytokines/chemokines in the lung tissues. *n* = 3–12. (E) Serum concentrations of IL-6, TNF-α, and CXCL10 at 4 dpi. *n* = 3–7. (F) Representative H&E images of lungs of mock control and Omicron BA.2 or Delta infected newly-weaned hamsters at 4 dpi. Scale bar = 500 µm. (G) Representative H&E images of NT, trachea, and lung of Omicron BA.2 or Delta infected newly-weaned hamsters at 4 dpi. Areas in the numbered squares were magnified, respectively. Scale bars = 500, 200, 50 µm. (H) Semiquantitative scores for histological changes in hamster lungs at 4 dpi. *n* = 5–14. Data represented mean ± SD. *p < 0.05, **p < 0.01, ***p < 0.001, ****p < 0.0001, by Two-way (A–D) or One-way (H) ANOVA.

Comparison between these two age groups challenged by SARS-CoV-2 showed that newly-weaned hamsters had more than one log lower viral load and infectious titre in both Omicron BA.2 and Delta infection with NT and lung at 4 dpi (Figure 1(A,B)), which indicate that both viruses replicate less effectively in newly-weaned hamsters than mature hamsters.

RT-qPCR profiling for cytokine/chemokine gene expression showed significantly higher basal mRNA expression levels of IFN-α, IFN-γ, and pro-inflammatory cytokine/chemokine IL-6, TNF-α, CXCL10, CCL2, and CCL3 in the lung of newly-weaned hamsters than mature hamsters before virus challenge (Figure 1(C)). A rapid upregulation of IL-6, TNF-α, IL-1β, IFN-γ, CXCL10, CCL2, and CCL3 was observed at 2 dpi in newly-weaned hamster lung tissues, which were significantly higher than their age-matched mock controls. The cytokines decreased back to basal level at 4 and 7 dpi, except for IL-1β and CXCL10. However, no IFN-α upregulation was detectable after Omicron BA.2 or Delta infection in newly-weaned hamsters (Figure 1(D)). On the contrary, mature hamsters had a significant increase in IFN-α expression together with most of the cytokine/chemokines from 2 to 7 dpi (Figure 1(D)). Serum concentration of IL-6, TNF-α, and CXCL10 were significantly higher in newly-weaned hamsters challenged by Omicron BA.2 or Delta virus than their age-matched mock controls (Figure 1(E)), indicating that systemic inflammatory responses were elicited by virus infection in newly-weaned hamster despite lower virus replication in respiratory tissues.

Histologically, Omicron BA.2-infected lung damages progressed from interstitial inflammatory infiltration at 2 dpi to diffuse alveolitis with severe alveolar space infiltration and exudation at 4 dpi same, which was similar to Delta infected lungs (Figure 1(F)), but BA.2 infected hamster lungs had significant lower histological score (Figure 1(H)). Importantly, some unique histopathological features were observed in Omicron BA.2 infected newly-weaned hamster, including severe submucosal oedema with eosinophil infiltration in nasal and tracheal mucosa, and severe perivascular oedema and with eosinophil infiltration in the lung tissue space around the large vessel and bronchi (Figure 1(G)). These histopathological changes were different from the dominant mononuclear cell infiltration induced by Delta variant and prototype SARS-CoV-2 infection [23].

### Brain involvement by Omicron BA.2 and Delta variants in newly-weaned hamsters after intranasal inoculation

**Figure 2.**
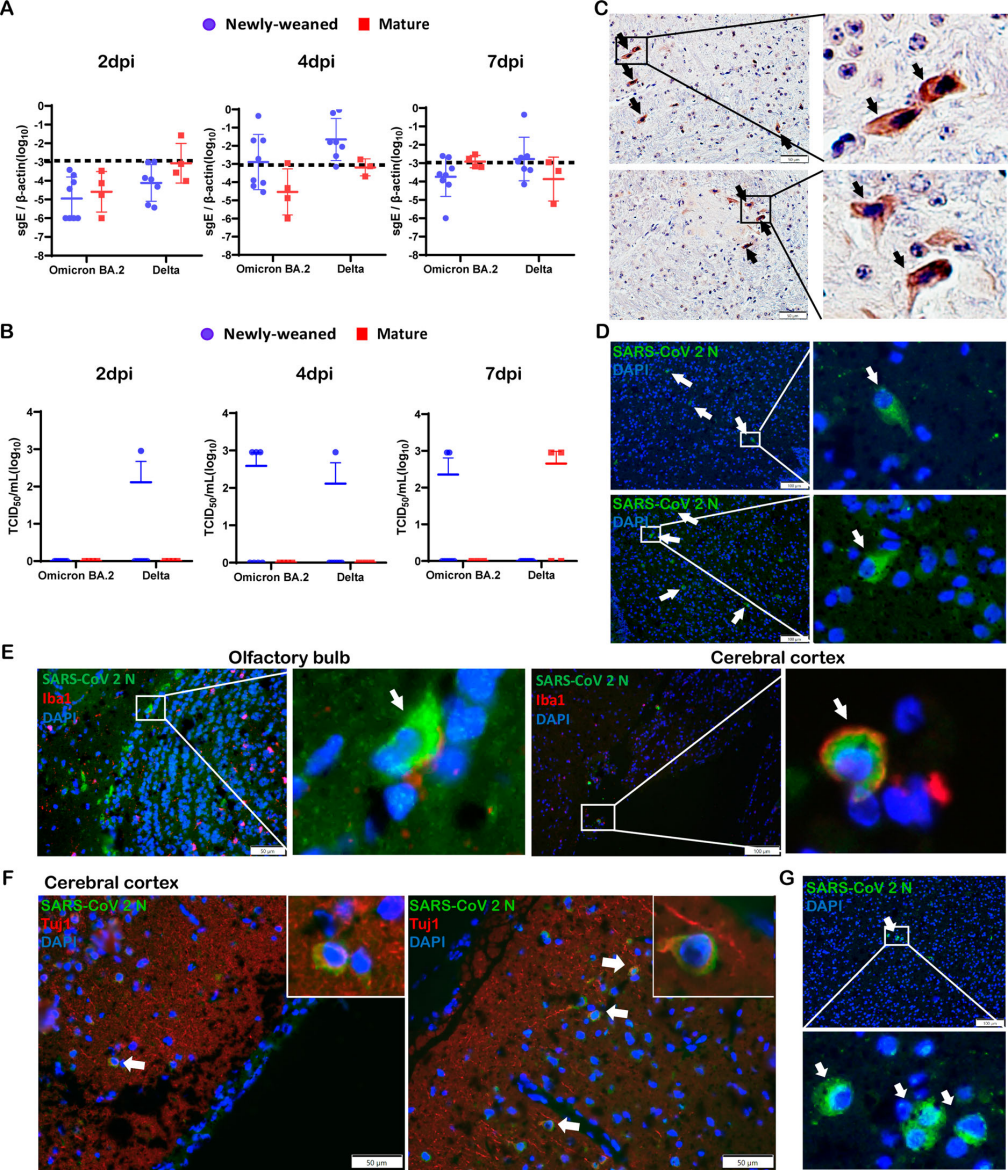
SARS-CoV-2 viral RNA and antigen expression in newly-weaned or mature hamster brain tissues after Omicron BA.2 or Delta virus infection. (A) Viral Sg-E gene copies in brain tissues. *n* = 3–8. (B) Infectious virus titre in brain tissues. *n* = 3–8. Data represented mean ± SD. Dashed lines indicated as detection limits. (C) Immunohistochemistry stained SARS-CoV-2 viral N protein in brain sections of Omicron BA.2 infected newly-weaned hamsters at 4 dpi. The positive cells in the boxed area were magnified and indicated by arrows. Scale bar = 50 µm. (D) Immunofluorescence stained SARS-CoV-2 viral N protein in brain sections of Omicron BA.2 infected newly-weaned hamsters at 4 dpi. N protein-positive cells stained in green fluorescence and indicated by arrows. The positive cells in the boxed area were magnified. Scale bar = 100 µm. (E) Double immunofluorescence stained N protein antigen (green) and microglial cellular marker Iba1 (red) in brain sections of Omicron BA.2 infected newly-weaned hamsters at 4 dpi. Iba1 and N antigen double-positive cells in boxed areas were magnified and indicated by arrows. Scale bar = 50  or 100 µm. (F) Double immunofluorescence staining of N antigen and neuron marker Tuj1 in brain tissue of Omicron BA.2 infected newly-weaned hamsters at 4 dpi. Tuj1 and N antigen double-positive cells in the insert. (G) Immunofluorescence stained N protein in brain tissue sections of Omicron BA.2 infected mature hamsters at 4 dpi. N protein-positive cells stained in green fluorescence and indicated by arrows. The positive cells in the boxed area were magnified. Scale bar = 50 µm (F&G).

SARS-CoV-2 subgenomic E gene was detected by RT-qPCR assays in the brain samples taken at 2, 4, and 7 dpi, altogether, 6/24 (25%) Omicron BA.2 and 11/21 (52.4%) Delta variant-infected newly-weaned hamster brains showed positive Sg-E, respectively (Figure 2(A)). Live virus was detected by TCID_50_ assay from 5/22 (22.7%) and 2/22 (9.1%) newly-weaned hamster brains after Omicron BA.2 and Delta variant infection, respectively (Figure 2(B)). In mature hamsters, subgenomic E gene were detected at lower frequency in Delta infection (3/10) when compared with newly-weaned hamster (Figure 2(A)); 2/11 Delta virus-infected mature hamsters had live infectious virus titre, while no live virus was detectable from Omicron BA.2-infected mature hamsters (Figure 2(B)). Brain tissues with positive viral RNA stained by SARS-CoV-2 spike RNA hybridization kit further confirmed the presence of virus gene inside the infected cells (Figure S1). Immunohistochemistry and immunofluorescence staining both showed sporadic viral N antigen-positive cells in the OB and cerebral cortex (Figure 2(C,D)). No signals were found in mock control brains. Double immunofluorescence staining for microglia cellular marker Iba1 and SARS-CoV-2 N antigen showed double-positive cells in the OB and cerebral cortex which indicated that microglial cells were infected (Figure 2(E)). N protein-expressing cells with positive neuronal marker Tuj1 were only rarely found (Figure 2(F)). In mature hamster brains, viral N-positive cells were also occasionally seen after Omicron BA.2 or Delta virus infection (Figure 2(G)). These findings indicated that intranasal challenge with SARS-CoV-2 Omicron BA.2 and Delta variants can infect the brain of hamsters. Live viruses were more frequently found in the brains of newly-weaned hamster infected by Omicron BA.2 than mature hamster.

### Pro-inflammatory cytokine/chemokine upregulation in the brain of newly-weaned hamster after intranasal inoculation of SARS-CoV-2 Omicron BA.2 and Delta variant

**Figure 3.**
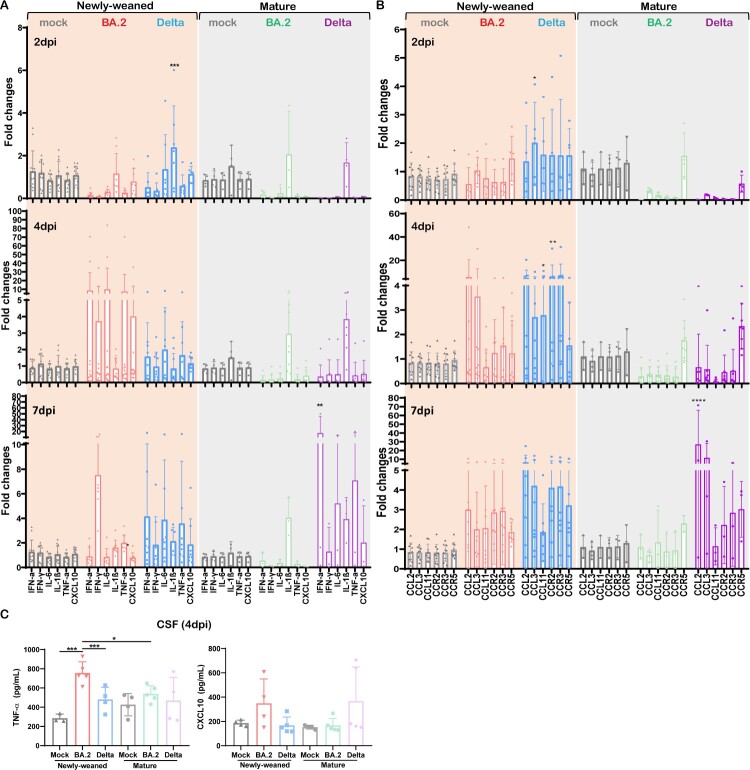
Pro-inflammatory cytokine/chemokine responses in brains of mock control, Omicron BA.2 or Delta virus-infected hamsters. (A) Relative mRNA expression levels of pro-inflammatory cytokines/chemokines in Omicron BA.2 or Delta infected newly-weaned or mature hamster brain tissues at 2, 4 and 7 dpi. *n* = 3–14. (B) Relative mRNA expression levels of CCL2, CCL3, CCL11, CCR2, CCR3, and CCR5 in the brain tissues. *n* = 3–12. (C) CSF concentrations of TNF-α and CXCL10 at 4 dpi. *n* = 3–5. Data represented mean ± SD. *p < 0.05, ***p < 0.001, ****p < 0.0001, by Two-way ANOVA.

Unlike the lung, the basal expression level of innate cytokines/chemokines in uninfected newly-weaned hamster brains was significantly lower than those in mature hamster, except for a similar level of IL-1β (Figure S2). At 4 dpi after Omicron BA.2 infection, newly-weaned hamster showed around 10-fold increase of IFN-α, IL-6, and TNF-α, and 4-fold increase of IFN-γ and CXCL10 in brain. Then all except IFN-γ decreased to the basal level at 7 dpi (Figure 3(A)). In the brains of mature hamsters, IL-1β was upregulated from 2 dpi through 7 dpi, IFN-α, IL-6, TNF-α, and CXCL10 were only upregulated at 7 dpi (Figure 3(A)). C–C motif chemokine 11 (CCL11) has been reported to be particularly associated with SARS-CoV-2-related brain damages by limiting neurogenesis [32]. As expected, we found that CCL11 together with CCL2, CCL3, and the receptors CCR2, CCR3, and CCR5 were all upregulated in newly-weaned hamster brain tissues after Delta variant infection at 2, 4, and 7 dpi, of which CCL11 and CCR2 were significantly higher at 4 dpi (Figure 3(B)). In Omicron BA.2 infected newly-weaned hamster brains, all these chemokines and receptors were upregulated at 4 and 7 dpi while not reaching statistically significant differences comparing with age-matched mock controls. In mature hamsters, only CCR5 was slightly upregulated at 2 and 4 dpi after Omicron BA.2 or Delta infections. Though most of these genes were upregulated at 7 dpi after Delta infection (Figure 3(B)), we did not see CCL11 upregulation in mature hamster brains after either Omicron BA.2 or Delta infection. In the CSF of newly-weaned hamsters, TNF-α concentrations were increased at 4 dpi after Omicron BA.2 infection (Figure 3(C)). Our findings suggested that higher pro-inflammatory cytokine/chemokine response occurred in the brains of newly-weaned hamsters after Omicron or Delta variant infection.

### Histopathological changes in brain tissues of the newly-weaned hamsters after intranasal challenge by Omicron BA.2 or Delta variant

**Figure 4.**
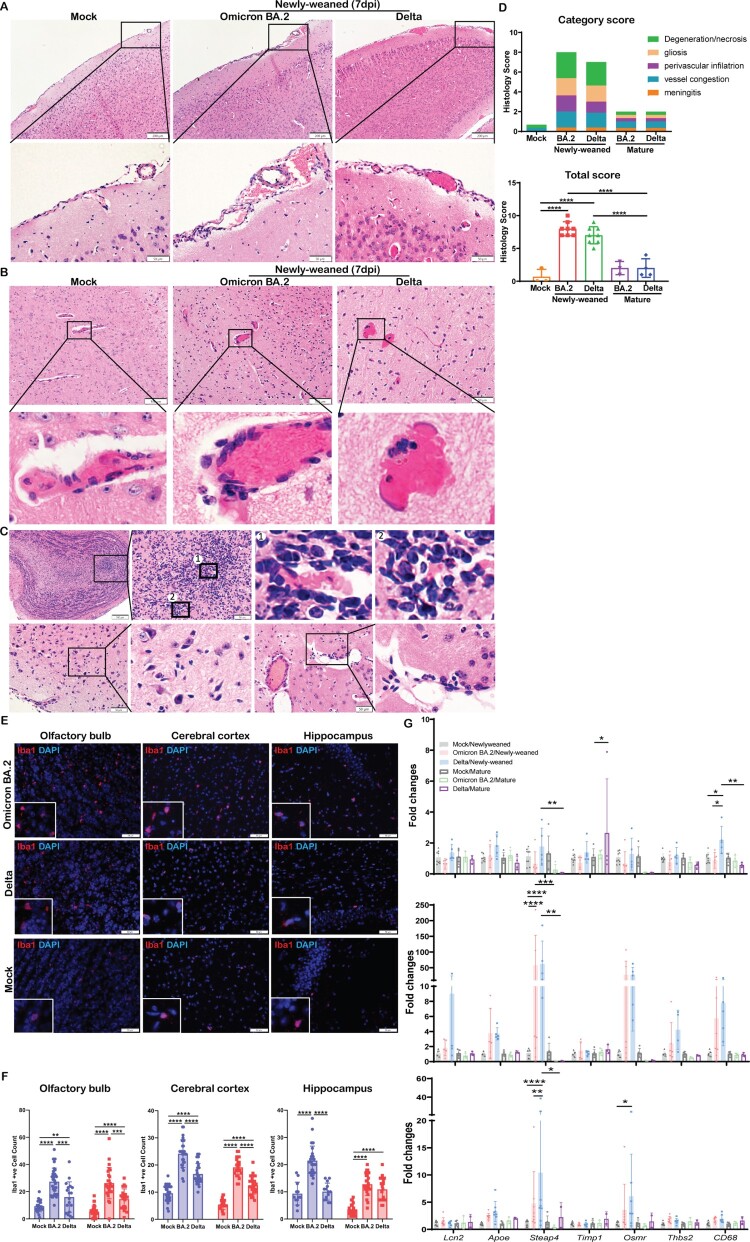
Histological changes of inflammation in the brain tissues of newly-weaned hamsters after Omicron BA.2 or Delta virus infection. (A) Representative H&E images of meninges of newly-weaned hamsters from mock control or SARS-CoV-2 infected hamsters. The meninges showed vascular congestion and inflammatory cell infiltration at 7 dpi after Omicron BA.2 or Delta virus infection. Boxed areas were magnified in the lower panel. Scale bar = 200 µm (upper panel), 50 µm (lower panel). (B) Representative H&E images of cerebral cortex from mock control (left) or SARS-CoV-2 infected newly-weaned hamsters. Cerebral cortex showed vascular congestion and increased cellularity around vessel wall (middle) and small haemorrhagic foci (right) at 7 dpi. Scale bar = 100, 50 µm. (C) The H&E images of OB of Omicron BA.2 infected newly-weaned hamsters at 7 dpi showed microglia accumulation around the blood vessel (upper panel). Images labelled with 1&2 are two boxed areas magnified. The images in the lower panel showed microgliosis in the cerebral cortex of Omicron BA.2 infected newly-weaned hamsters at 7 dpi. Scale bar = 50 µm. (D) Semiquantitative scores for histological changes in hamster brains. The scores for each category of brain histological changes (upper) and the total scores were presented (lower). *n* = 3–8. (E) Representative images of immunofluorescence stained microglia with Iba1 antibody in OB, cerebral cortex and hippocampus of Omicron BA.2 or Delta infected newly-weaned hamster brains at 7 dpi. Microglia were stained red in the brain parenchyma. Magnified Iba1-positive cells in the insert. Scale bar = 50 µm. (F) The number of Iba1-positive microglia in brain sections in different anatomical regions obtained by quantitative image analysis. 10–20 microscopic fields at 400× magnification from different anatomic regions were counted for each sample. *n* = 3–4. (G) Relative mRNA expression levels for genes regulating activation of microglia and astrocytes in Omicron BA.2 or Delta infected newly-weaned or mature hamster brains at 2, 4, and 7 dpi. *n* = 3–8. Data represented mean ± SD. *p < 0.05, **p < 0.01, ***p < 0.001, ****p < 0.0001 when comparing with mock control by One-way (D&F) or Two-way ANOVA (G).

No gross pathology such as cerebral oedema or haemorrhage was observed in any of the hamsters at 2, 4, or 7 dpi after infection. Paraffin-embedded brain tissues were cut into sagittal sections and stained by H&E for histological examination. Anatomical regions including OB, cerebral cortex, hippocampus, midbrain, thalamus, cerebellum, pons, and medulla were assessed (Figure S3). Firstly, meningeal congestion and enlarged vascular space with some inflammatory cell infiltration suggestive of mild meningitis were frequently observed in newly-weaned hamsters at 4 dpi by Omicron BA.2 or Delta virus challenge, but less frequently at 7 dpi (Figure 4(A)). In the brain parenchyma, congestion of the small blood vessels and capillaries with enlarged vascular space and perivascular inflammatory cells infiltration were frequently observed and small foci of haemorrhage were occasionally seen in newly-weaned hamsters challenged by Omicron BA.2 or Delta virus at 4 and 7 dpi (Figure 4(B)). Furthermore, increased microglia were observed which mainly affected OB, olfactory cortex, and midbrain cortex in most of the newly-weaned hamsters (Figure 4(C)). The brain of infected mature hamsters showed increased microglia while the meningeal and brain parenchymal vascular inflammatory histological changes were mostly absent. Comparison of the frequency and severity of the histopathological changes by semiquantitative scoring showed that Omicron BA.2 and Delta-infected newly-weaned hamsters had significantly higher scores of meningitis, parenchymal gliosis, and vascular congestion compared to mock controls (Figure 4(D), Supplementary Table 3). Furthermore, immunofluorescence staining of microglial cellular marker Iba1 and image quantification showed a significant increase of Iba1-positive cell in the newly-weaned hamster brains at 7 dpi (Figure 4(E,F)). Though infected mature hamsters also showed an increase in Iba1-positive microglial cells compared to their age-matched controls (Figure 4(F)), determination of the status of microglial cell activation by real-time RT-qPCR only showed significant upregulation of *Lcn2* (Lipoclin-2), *Steap4* (six transmembrane epithelial antigen of prostate-4), *Timp1*(TIMP metallopeptidase inhibitor 1), *Osmr* (Oncostatin M Receptor), *Thbs2* (Thrombospondin 2), and *Apoe* (Apolipoprotein E) in brain tissues of newly-weaned hamster at 4 and 7 dpi, but not in mature hamsters (Figure 4(G)). These findings strongly indicated that a higher level of brain microglial cell activation was induced by SARS-CoV-2 infection of newly-weaned hamsters than mature hamster.

### Neuronal degeneration, apoptosis, and necrosis in brain tissues of newly-weaned hamster after Omicron BA.2 or Delta infection

**Figure 5.**
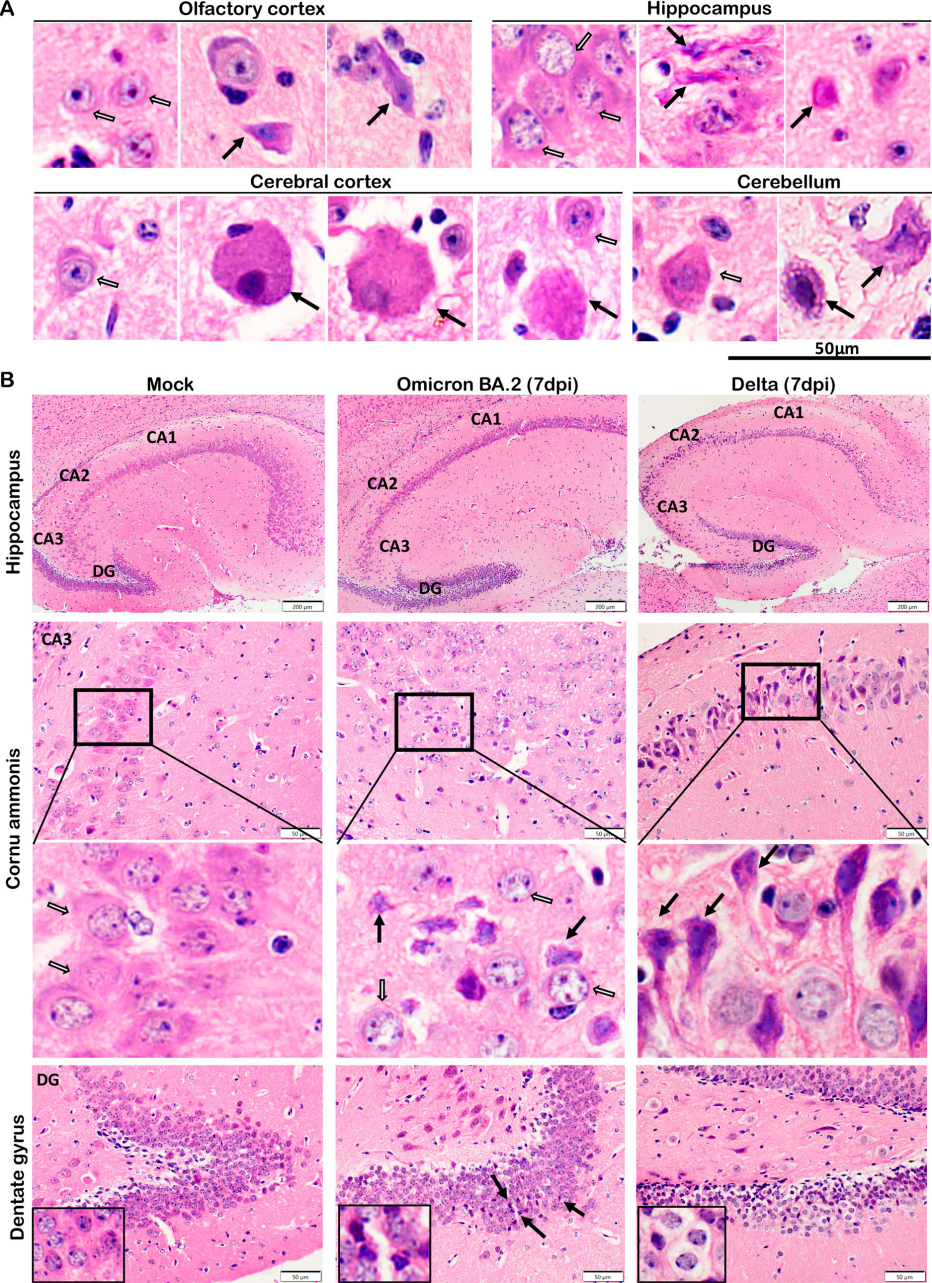
Neuron degenerative and necrotic changes in the hippocampus and DG of newly-weaned hamsters infected by Omicron BA.2 or Delta virus at 7 dpi. (A) Representative images with high magnifications showing the neuron degenerative changes (solid arrows) frequently observed in the brains of Omicron BA.2 infected newly-weaned hamsters at 4 or 7 dpi. Open arrows indicated normal neurons in olfactory cortex, hippocampus, cerebral cortex, and cerebellum, respectively. Scale bar = 50 µm. (B) Representative images of hippocampus of mock, Omicron BA.2 or delta virus-infected newly-weaned hamsters at 7 dpi. The anatomical sub-regions of hippocampus, cornu ammonis 1, 2, 3 (CA1, CA2, CA3) and DG sub-regions of the hippocampus are labelled. Mock control hamster hippocampus showed normal morphology of neurons in the CA3 and DG sub-regions (open arrows). At 7 dpi, among the normal neurons (open arrows), degenerative and necrotic pyramidal neurons were shown as dark coloured, shrunk cell body with pyknotic nuclei (solid arrows); the DG area of infected hamsters showed neuron degeneration with dark coloured, shrunken cell body in Omicron BA.2 infected newly-weaned hamster, and also vacuolated degeneration in Delta infected newly-weaned hamster (magnified degenerative neurons in the insert) Scale bar = 200, 50 µm.

**Figure 6.**
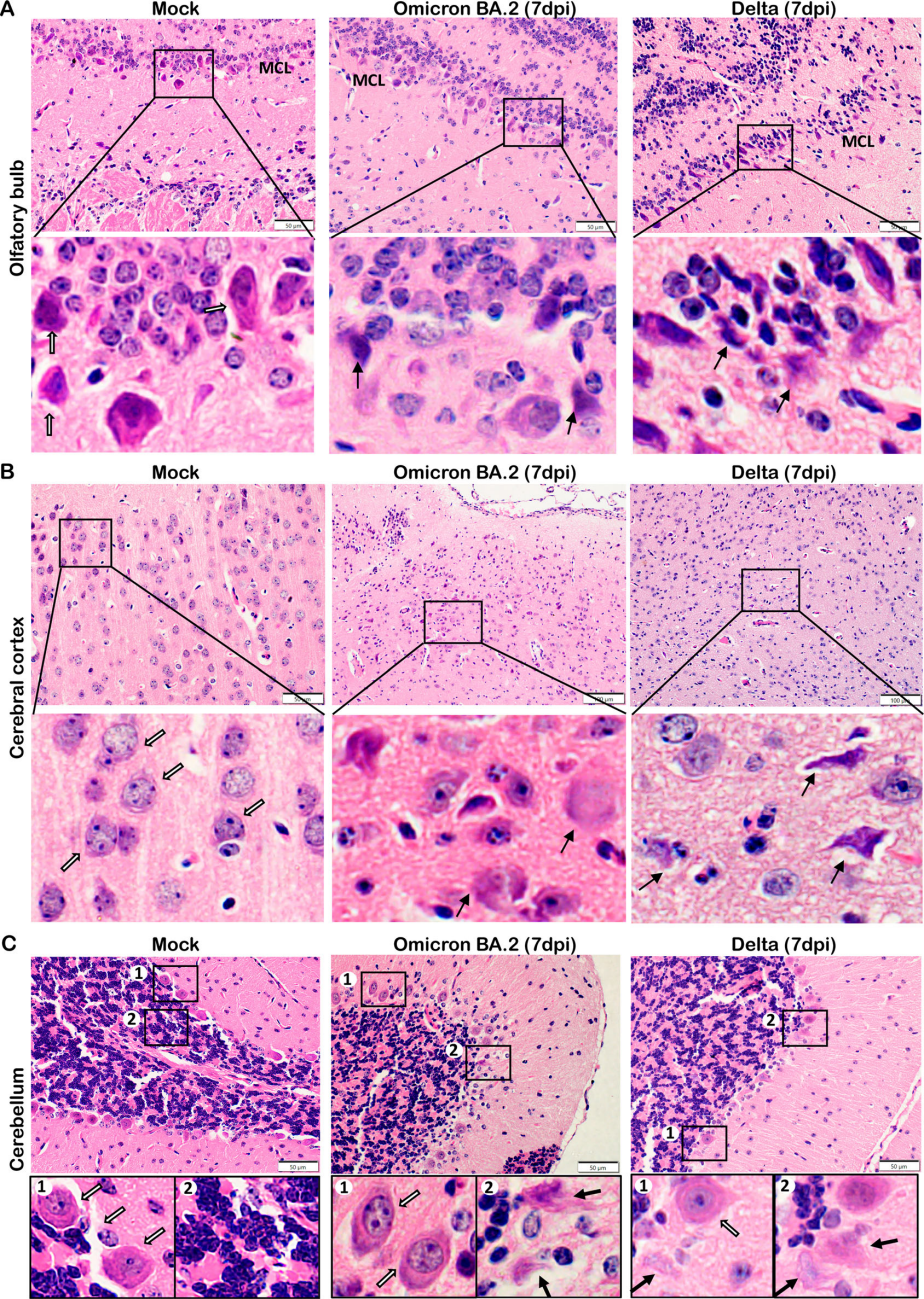
Neuron degenerative and necrotic changes in the OB, cerebral cortex and cerebellum of newly-weaned hamsters infected by Omicron BA.2 or Delta virus at 7 dpi. (A) Representative images of OB (OB) magnified to show the mitral cell layer (MCL) in mock, Omicron BA.2, or Delta virus-infected newly-weaned hamsters at 7 dpi. Further magnified MCL in squared areas showed normal mitral cells with pyramidal shape, abundant cytoplasm and vesicular nuclei in mock control hamster (open arrows), and the cluster of small sized granule nerve cells in round shape with scanty cytoplasm and dark nuclei. While in infected newly-weaned hamster brain, degenerated neurons in MCL showed dark and shrunken nuclei (solid arrows) and accumulated glial cells. Scale bar = 50 µm. (B) Representative images of cerebral cortex of mock control, Omicron BA.2 or Delta virus-infected newly-weaned hamsters at 7 dpi. In mock control brain, the normal morphology of neurons was indicated by open arrows in a magnified image of the squared area. In Omicron BA.2 or Delta infected newly-weaned hamster brain, pyramidal neurons showed degenerative changes with ballooning cell body and loss of nuclei (arrows in Omicron BA.2, 7 dpi); some degenerative neurons showed eccentric shape, dark and shrunken nuclei (solid arrows, in Delta 7 dpi). Scale bar = 50 or 100 µm. (C) Representative images of H&E stained hamster cerebellum. Normal morphology of molecular layer and granular layers were shown in the mock controls at lower magnification. Magnified images of granular layer in the squares showed normal granule cells and Purkinje neurons (open arrows). The cerebellum of hamster infected by Omicron BA.2 or Delta virus at 7 dpi showing a some Purkinje cells degeneration (solid arrows) among the normal Purkinje cells (open arrows). Scale bar = 50 µm.

**Figure 7.**
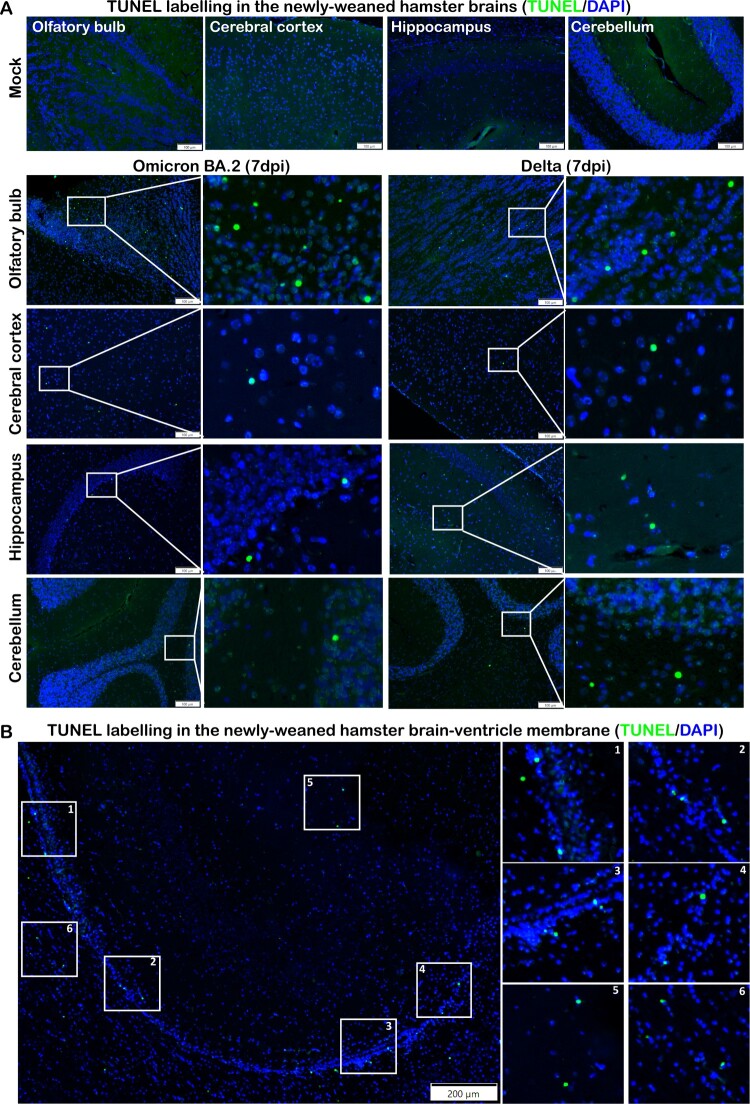
TUNEL staining in the brain tissues of newly-weaned hamsters infected by Omicron BA.2 or Delta virus at 7 dpi. (A) Representative images of different brain structure stained by TUNEL. Mock control hamster brain, from left to right, OB, cerebral cortex, hippocampus, and cerebellum showed no TUNEL labelled cells. TUNEL staining images of Omicron BA.2 (left two panels) and Delta (right two panels) infected newly-weaned hamsters brain tissue at 7 dpi showing TUNEL-positive cells (in green). Positive cells in the squared area were magnified and shown on the right. Scale bar = 100 µm. (B) Representative images of increased TUNEL-positive ependymal cells lining the lateral ventricle in newly-weaned hamster brain after Omicron BA.2 infection at 7 dpi. Positive cells in numbered squared areas were magnified and shown on the right. Scale bar = 200 µm.

Neuronal degenerative changes in response to inflammation were common and could result in chronic neurological complications. These changes were frequently identified in H&E stained brain sections from multiple locations of newly-weaned hamsters at 4 and 7 dpi. The degenerating neurons were characterized by hypereosinophilic and ballooning cytoplasm and shrunken nucleus with nuclear dissolution (Figure 5(A)). In the hippocampus regions, the pyramidal neurons in the Cornu Ammonis (CA) regions had eccentric shape with shrunken nuclei clustered in the CA1 to CA 3 region. Similar degenerative changes were also found in the Dentate Gyrus region (DG) (Figure 5(B)). In the OB, midbrain cortex, and occasionally cerebellum, degenerative neurons were also observed, with occasional necrotic neurons (Figure 6). TUNEL-positive cells were more abundant in the OB but also occasionally found in the cerebral cortex, hippocampus, and cerebellum cortex and in the ependymal epithelium of ventricles (Figure 7). In the brain of mature hamsters, the degenerating neurons were found mainly confined in the hippocampus across CA1-CA3 regions at 7 dpi (Figure S4, Supplementary Table 3). TUNEL-positive cells were rarely found in the brain of mock controls.

### SARS-CoV-2 Omicron BA.5 variant causes similar histopathological changes as BA.2 in the lung and brain of newly-weaned hamsters

**Figure 8.**
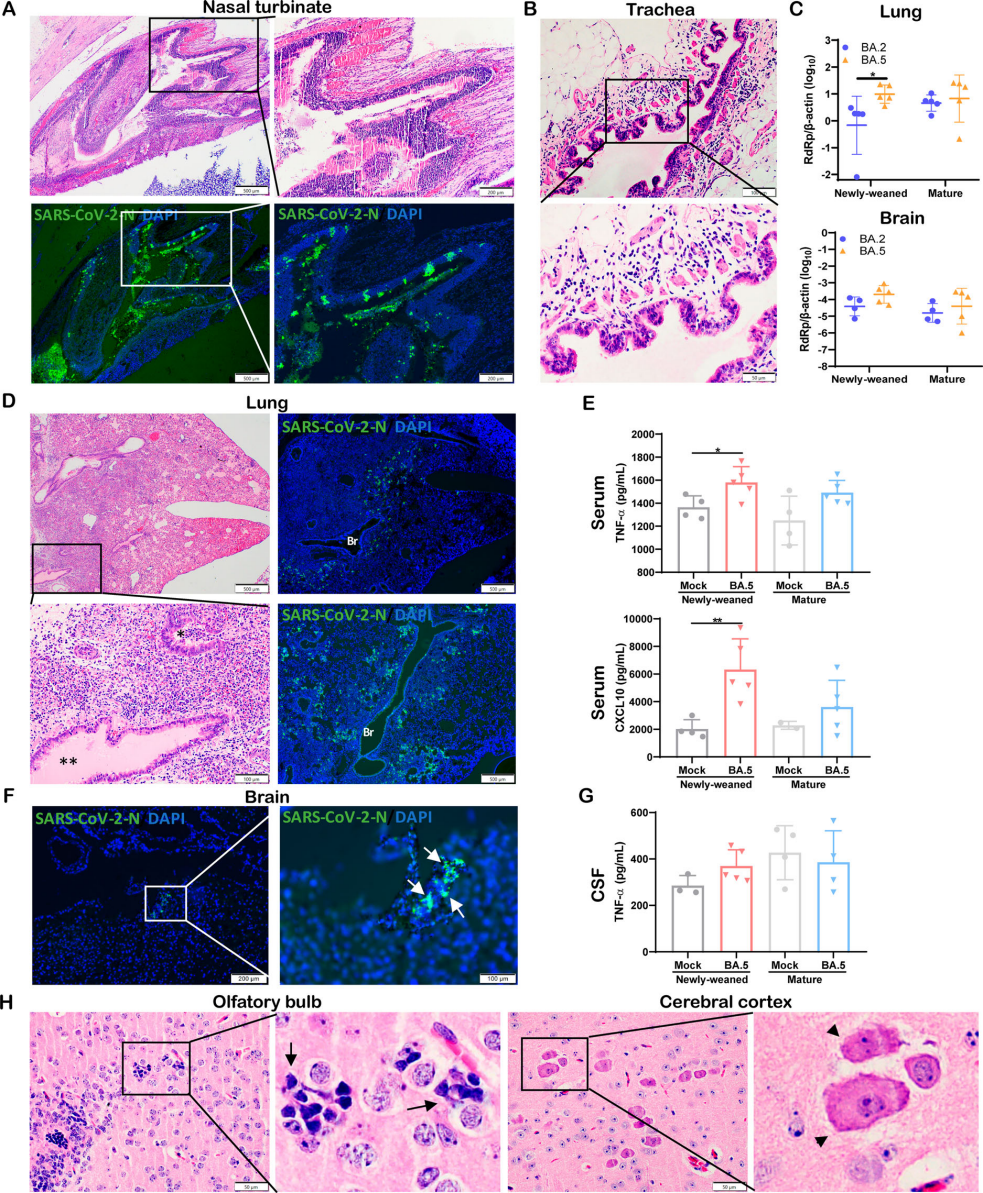
Omicron BA.5 infection in the respiratory and brain tissues of newly-weaned hamsters at 4 dpi. (A) Representative H&E images (upper panel) and immunofluorescence stained SARS-CoV-2 N protein images (lower panel) of NT after Omicron BA.5 infection of newly-weaned hamsters. Scale bar = 500, 200 µm. (B) Representative H&E images of trachea tissue sections of Omicron BA.5 infected newly-weaned hamster showing submucosal tissue oedema and inflammatory infiltrate including eosinophils infiltration. Scale bar = 100, 50 µm. (C) Viral RdRp gene copies in homogenized lung tissues or brain tissues of Omicron BA.2 or BA.5 infected newly-weaned and mature hamsters at 4 dpi. *n* = 5. Data represented mean ± SD. *p < 0.05, by Two-way ANOVA. (D) Representative images of H&E the lung tissues of Omicron BA.5 infected newly-weaned hamsters at 4 dpi. Squared area was magnified showing abundant secretion filling the lumen of bronchiole (**). The immunofluorescence stained images showing SARS-CoV-2 N antigen expression in the lung of BA.5 infected newly-weaned hamsters. “Br” indicates bronchiolar section. Scale bar = 500, 100 µm. (E) Serum concentrations of TNF-α and CXCL10 of mock or BA.5 infected hamsters at 4 dpi. Data represented mean ± SD. *n* = 4–5. Data represented mean ± SD. *p < 0.05, **p < 0.01 by One-way ANOVA. (F) Images of immunofluorescence stained viral N protein in the brain of BA.5 infected newly-weaned hamster at 4 dpi showing N antigen expression at the epithelium of circumventricular organ (arrows in magnified image). Scale bar = 200, 100 µm. (G) CSF concentrations of TNF-α in Omicron BA.5 infected hamsters at 4 dpi or mock controls. *n* = 4–5. (H) Representative H&E images of brain tissue sections of Omicron BA.5 infected newly-weaned hamsters at 4 dpi showing microgliosis in OB (arrows in magnified image) and neuron degeneration in cerebral cortex (arrowheads in magnified image). Scale bar = 50 µm.

SARS-CoV-2 Omicron subvariants continue to emerge. To investigate whether they may cause different pathology in newly-weaned hamsters, we compared their virological and histological features after intranasal challenge. The same intranasal dose of BA.5 or BA.2 caused a similar degree of nasal epithelial infection and tissue destruction (Figure 8(A)), and severe submucosal oedema with eosinophil infiltration in trachea (Figure 8(B)). In the newly-weaned hamster lungs, BA.5 infection had a significantly higher viral load than BA.2 at 4 dpi (Figure 8(C)). Lung tissue sections showed extensive viral N protein expression in alveolar epithelium, diffuse alveolar inflammatory cell infiltration, and protein-rich fluid exudation which also filled the bronchial lumen (Figure 8(D)). Significantly increased serum concentrations of TNF-α and CXCL10 in newly-weaned hamsters indicated severe systemic inflammation (Figure 8(E)). In the brain of newly-weaned hamsters, BA.5 infection also showed a higher viral load than BA.2 infection (Figure 8(C)) with occasionally observed viral N antigen (Figure 8(F)). Increased CSF concentrations of TNF-α (Figure 8(G)), parenchymal microgliosis, and neuronal degeneration or necrosis were frequently observed (Figure 8(H)).

## Discussion

The Syrian hamster SARS-CoV-2 infection model has been widely used for the studying the pathogenesis, antiviral therapy, and vaccination for SARS-CoV-2 infection [23,29,33–36]. To simulate SARS-CoV-2 infection at young age, we performed intranasal challenge of newly-weaned hamster with SARS-CoV-2 Omicron subvariants BA.2 and BA.5, and Delta variant. We demonstrated that they commonly induced brain parenchymal inflammation and neuronal degeneration in newly-weaned hamsters compared to mature hamsters.

In lung tissues, Omicron BA.2 replicated less effectively and produced a significantly lower viral load in newly-weaned hamsters than mature hamsters. However, the histopathological damages in the lungs were similar, which is manifested as diffuse alveolitis at 4 dpi. In paediatric COVID-19 patients, lower viral load in respiratory specimens than that of adult patients was attributed partly to the higher basal expression and activity of interferon in the respiratory epithelium of children [37], and to some degree of cross-protection from natural infection by other human coronaviruses [38]. Consistent with findings in children, the basal expression levels of interferon-α and many pro-inflammatory cytokine/chemokines were significantly higher in lungs of newly-weaned hamsters than mature hamsters before infection. However, we detected no further induction of IFN-α after virus infection despite a surge of pro-inflammatory responses (IL-6, IL-1β, TNF-α, IFN-γ CCL2, CCL3, and CXCL10) compared to mature hamsters. This may suggest that the first-line antiviral response by interferon could not be further activated in the lungs of newly-weaned hamsters. Other host factors such as the amount of receptor expression in the lungs may also contribute to the lower viral load in young hamsters [39].

Despite the lower viral load in their lung tissue, infected newly-weaned hamsters commonly developed brain inflammatory damages regardless of the SARS-CoV-2 variants used for challenge. At 4 dpi, pro-inflammatory cytokines/chemokines including IFN-α, were substantially upregulated in the brain tissue of newly-weaned hamsters, accompanied by histological changes of meningeal inflammation, vascular congestion in brain parenchyma, increased perivascular space and cellularity, and small foci of haemorrhage.

In mature hamster brain, only IL-1β was mildly upregulated at 2 and 4 dpi after Omicron BA.2 and Delta virus challenge. Pro-inflammatory response by gene expression of IFN-α, IL-6, and TNF-α responses were not detected until 7 dpi after Delta infection but not after Omicron infection. Histological changes of inflammation in brain tissue were limited to vascular congestion with enlarged vascular space in brain parenchyma without inflammatory cell infiltration or meningitis.

While the brain is an immune-privilege site, inflammation in response to a variety of endogenous and exogenous insults would lead to neuronal injury initiated by microglia, which are the resident macrophages of the central nervous system. Proliferation, accumulation, and activation of microglia are common features of brain parenchymal inflammation, which could be both beneficial and detrimental. In brain tissues of SARS-CoV-2 infected newly-weaned hamster, we observed increased number of microglial cells widely distributed in OB, cerebral cortex, and hippocampus. In some cases, the microglial cells clustered together to form microgliosis [40,41]. Several genes (*CD68, Lcn2, Apoe, Steap4, Timp1, Osmr*, and *Thbs2*) related to the activation status of microglial cells [42,43] were significantly upregulated as demonstrated by RT-qPCR assay. The most prominent upregulated cytokine/chemokine genes including IL-6, TNF-α, CCL2, and CCL3 in the brain tissue at 4 dpi could also be attributed to microglial activation [44,45]. Activation of microglial cells leads to further release of inflammatory mediators, increased blood–brain barrier permeability, and recruitment of systemic inflammatory cells into the brain, which could perpetuate inflammatory damages [45]. Consistent with these findings, TNF-α concentration in the CSF increased significantly in newly-weaned hamster infected by Omicron or Delta virus. In mature hamster, microgliosis was also observed in a small percentage of brain samples and mostly localized in OB and olfactory cortex without upregulation of those genes of microglial activation. These findings suggested that the olfactory mucosa may be one of the sites of virus entry and also a better immune regulatory control of brain microglial responses in the mature than newly-weaned hamsters [46]. Degenerating neurons were distributed in multiple anatomical regions including OB, olfactory cortex, cerebral cortex, hippocampus, and cerebellum which suggested different types of neurons were affected. Consistent with the minimal pro-inflammatory cytokine responses in brains of mature hamsters, the histological changes of inflammation and degeneration were confined to the OB, but in some animals also in the olfactory cortex and hippocampus.

Up to date, evidence for direct SARS-CoV-2 invasion into the brain has been controversial. This uncertainty is partly attributed to the different sensitivity and specificity of virus detection methods. To better understand the scope of SARS-CoV-2 virus infection of brain cells at young age, we studied the hamsters infected by different virus strains using RT-qPCR for viral load, innate immune gene expression profile, *in situ* hybridization, immunohistochemistry, and histological examination. Our RT-qPCR for subgenomic E gene confirmed the presence of replicating virus in the brain albeit at low level. RNAScope using spike gene-specific probe demonstrated viral spike RNA in brain cells of newly-weaned hamsters. Immunohistochemistry and immunofluorescence staining for viral N protein further confirmed the infection of microglia and a few neurons. Most importantly, live viruses were detected from 15.9% (7/44) of infected newly-weaned hamster brains, and 9.1% (2/22) of infected mature hamster brains. Taken together, we conclude that intranasal virus inoculation of SARS-CoV-2 Omicron and Delta variant can infect and replicate in brain tissue of newly-weaned hamsters at limited scale, but more frequently than that of mature hamsters. Consistent with our findings, previous *in vitro* study of brain organoids reported that SARS-CoV-2 virus can infect human microglia and neuron [47,48]. While virus entry through the olfactory mucosa to the brain is one possible route, the immature blood–brain barrier of the developing brain in newly-weaned hamster which is weakened or damaged by the systemic inflammation induced by the SARS-CoV-2 pneumonia is another possible route [46]. Other studies on the mechanisms of SARS-CoV-2 associated neurological symptoms in mature C57BL/6 mice and rats reported that administration of spike protein S1 subunit to the brain or hippocampus area induced microglia activation and neuron cell death [49,50]. These reports tend to support the conclusion that direct virus infection of brain cells is not essential in inducing brain damage.

Though Omicron BA.2 and BA.5 both replicate less effectively in respiratory tissue of newly-weaned hamsters comparing to Delta virus, BA.5 replicated more effectively than BA.2. BA.2 and BA.5 caused diffuse alveolitis and more severe submucosal eosinophil infiltration and submucosal oedema in upper respiratory tract. This may account for, at least in part, the symptoms of croup in young children infected by the Omicron virus [51]. Infections by other respiratory viruses such as parainfluenza, influenza A and B, respiratory syncytial virus, and adenovirus can often cause croup in young children [52]. Since the subglottic structure is the narrowest part of a child's upper respiratory tract, infection of the upper airway causing inflammation, mucosal and submucosal oedema may lead to life threatening airway obstruction [52,53]. Taken together, SARS-CoV-2 Omicron subvariants can still cause respiratory or brain complications in young children. Vaccination should be given to provide protection against possible complications.

One limitation of our study is that the longest period of study was 7 days after virus infection. We are not able to provide information on whether these young hamsters could completely recover when they get mature, or how long the brain damages can persist. It has only been 3 years since the COVID-19 pandemic started, clinical studies showed that neurological symptom can persist for a period of 6 months after recovery from the infection [11]. Close follow-up studies are needed to understand the duration of CNS symptoms in human and animal models with or without specific treatments.

## Supplementary Material

Supplemental Material
