# Supplementary material for: Intranasal infection by SARS-CoV-2 Omicron variants can induce inflammatory brain damage in newly weaned hamsters
Source: Emerg Microbes Infect. 2023 Jun 12;12(1):2207678. doi: 10.1080/22221751.2023.2207678 (PMC10262819; doi:10.1080/22221751.2023.2207678)
Supplement: Supplemental Material [file TEMI_A_2207678_SM2947.zip › Supplementary Tables 230126.docx]

**Supplementary Table 1. primers and probes for real time RT-PCR detection of viral load and mRNA expression of hamster cytokines/chemokine genes.**

| **Gene name** | **Forward primer (5’ to 3’)** | **Reverse Primer (5’ to 3’)** |
| --- | --- | --- |
| *SARS-CoV-2 RdRp* | CGCATACAGTCTTRCAGGCT | GTGTGATGTTGAWATGACATGGTC |
|  | Probe (5’ to 3’): FAM-TTAAGATGTGGTGCTTGCATACGTAGAC-lABkFQ | |
| *SARS-CoV-2 SgE* | CGATCTCTTGTAGATCTGTTCTC | ATATTGCAGCAGTACGCACACA |
|  | Probe (5’ to 3’): FAM-ACACTAGCCATCCTTACTGCGCTTCG-BBQ | |
| *β-actin* | ATGGCCAGGTCATCACCATTG | CAGGAAGGAAGGCTGGAAAAG |
|  | Probe (5’ to 3’): Cy5-AGCGGTTCCGTTGCCCTGAG-IABkFQ | |
| *IL-1β* | GTGGACAACAAAGCTCGTGG | AGCCCGTCAACCTCAAAGAA |
| *IL-6* | TGTCTTCTTGGGACTGCTGC | CCAAACCTCCGACTTGTTGA |
| *TNF-α* | CACCCACCGTCAAGGATTCA | TTGGCTGGGCAATGAAGAGT |
| *IFN-α* | AGACTGGGAGTTGCCTGTGA | GAGGAATCCAGGGCTTTCCAG |
| *IFN-γ* | ATGGAGGGGACCTCGTCTTT | GATGGCCTGGTTGTCCTTCA |
| *CXCL10* | TACGTCGGCCTATGGCTACT | TTGGGGACTCTTGTCACTGG |
| *CCL3* | GGTCCAAGAGTACGTCGCTG | GAGTTGTGGAGGTGGCAAGG |
| *CCL2* | AGGCCAAGAAGGAACGAGC | AGGGCTAAAAGAGGAAGGCAG |
| *CCL11* | TGCACCCTGAGAGCCATAGT | TGGGAGGATTTGGTCCAGGT |
| *CCR2* | ATCACTTAGACCAGGCCATGC | GGGCACTGTTTGCAGAGACG |
| *CCR3* | CCCATCAACTGGGGAGCAAG | TGAATGGGGTCATTGCGACT |
| *CCR5* | ATCACCTCTCAGACATCCGT | TTTACATCGGCTTTTCGGCAG |

| **Gene name** | **Forward primer (5’ to 3’)** | **Reverse Primer (5’ to 3’)** |
| --- | --- | --- |
| *Lcn2* | CCGAGGCCCAAAGAAGTTTCA | AGCAGGATGGAGGTGACATTG |
| *Steaps4* | TGATTCCTACTTGGCGCTGG | TCCAGTTGACCGTGTTGCTT |
| *Timp1* | GCATACTGCAACTCCGACCT | CCGCATCGAATCCTTTGAGC |
| *Osmr* | CAGGACATGCCAGCTACAGA | TCATAGCAGGTGACTGGGGA |
| *Thbs2* | ACAACGATGGTGTGAGCGAT | TGTTGTCCGTGTCGATCTGG |
| *Apoe* | CCGCTCAGAATCAGGACGAT | CATGCCAGCTGCTCGTTTAC |
| *CD68* | CAAGGGGGCTCTTGGAAACT | AATATAGCGATGCCCCAGGC |

**Supplementary Table 2. primers for real time RT-PCR determination of glial cell activation related genes.**

**Supplementary Table 3. Frequency of histopathological changes observed in hamster brain at 4 & 7dpi after Omicron BA.2 or Delta virus intranasal inoculation**

|  | Newly-weaned | | | | Mature | | | |
| --- | --- | --- | --- | --- | --- | --- | --- | --- |
|  | Omicron BA.2 | | Delta | | Omicron BA.2 | | Delta | |
|  | 4dpi | 7dpi | 4dpi | 7dpi | 4dpi | 7dpi | 4dpi | 7dpi |
| No. of Samples ^a^ | **15** | **8** | **14** | **7** | **10** | **7** | **10** | **7** |
| Meningitis^b^ (%) | 11(73.3)*** | 3(37.5) | 12(85.7)**** | 3(42.9) | 0(0) | 0(0) | 0(0) | 0(0) |
| Vasculature Congestion^b^ (%) | 10(66.7) | 6(75.0) | 11(78.6) | 3(42.9) | 5(50.0) | 4(57.1) | 5(50.0) | 2(28.6) |
| Perivascular infiltration^b^ (%) | 5(33.3) | 2(25.0) | 4(28.6) | 2(28.6) | 0(0) | 0(0) | 3(30.0) | 0(0) |
| Gliosis ^b^ (%) | 10(66.7) | 6(75.0) | 8(57.1) | 4(57.1) | 3(30.0) | 2(28.6) | 4(40.0) | 3(42.9) |
| Neuron degeneration^b^ (%) | 10(66.7) | 7(87.5) | 11(78.6) | 5(71.4) | 9(90.0)^c^ | 6(85.7) ^c^ | 8(80.0) ^c^ | 5(71.4) ^c^ |

a. Number of brains sections examined

b. Number of sections showing the histopathological changes

c. Number and percentage of hippocampus neuron degeneration observed

***p<0.001, **** p<0.0001, comparing with mature hamsters infected with same virus strain at same dpi by Fisher’s exact test.
