# Supplementary figures and images for: Intranasal infection by SARS-CoV-2 Omicron variants can induce inflammatory brain damage in newly weaned hamsters
Source: Emerg Microbes Infect. 2023 Jun 12;12(1):2207678. doi: 10.1080/22221751.2023.2207678 (PMC10262819; doi:10.1080/22221751.2023.2207678)

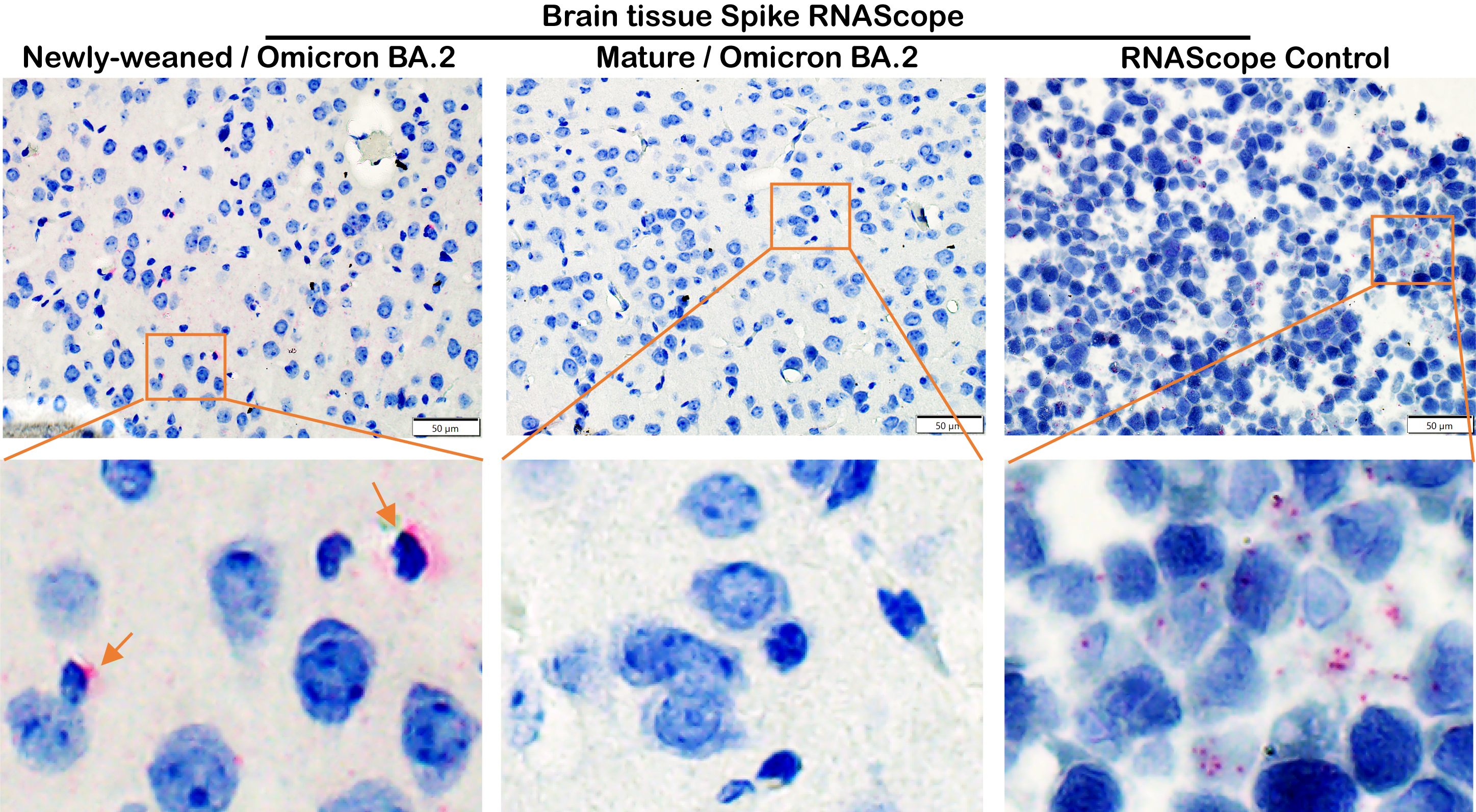

Supplement: Supplemental Material [file TEMI_A_2207678_SM2947.zip › Fig S1.tif]

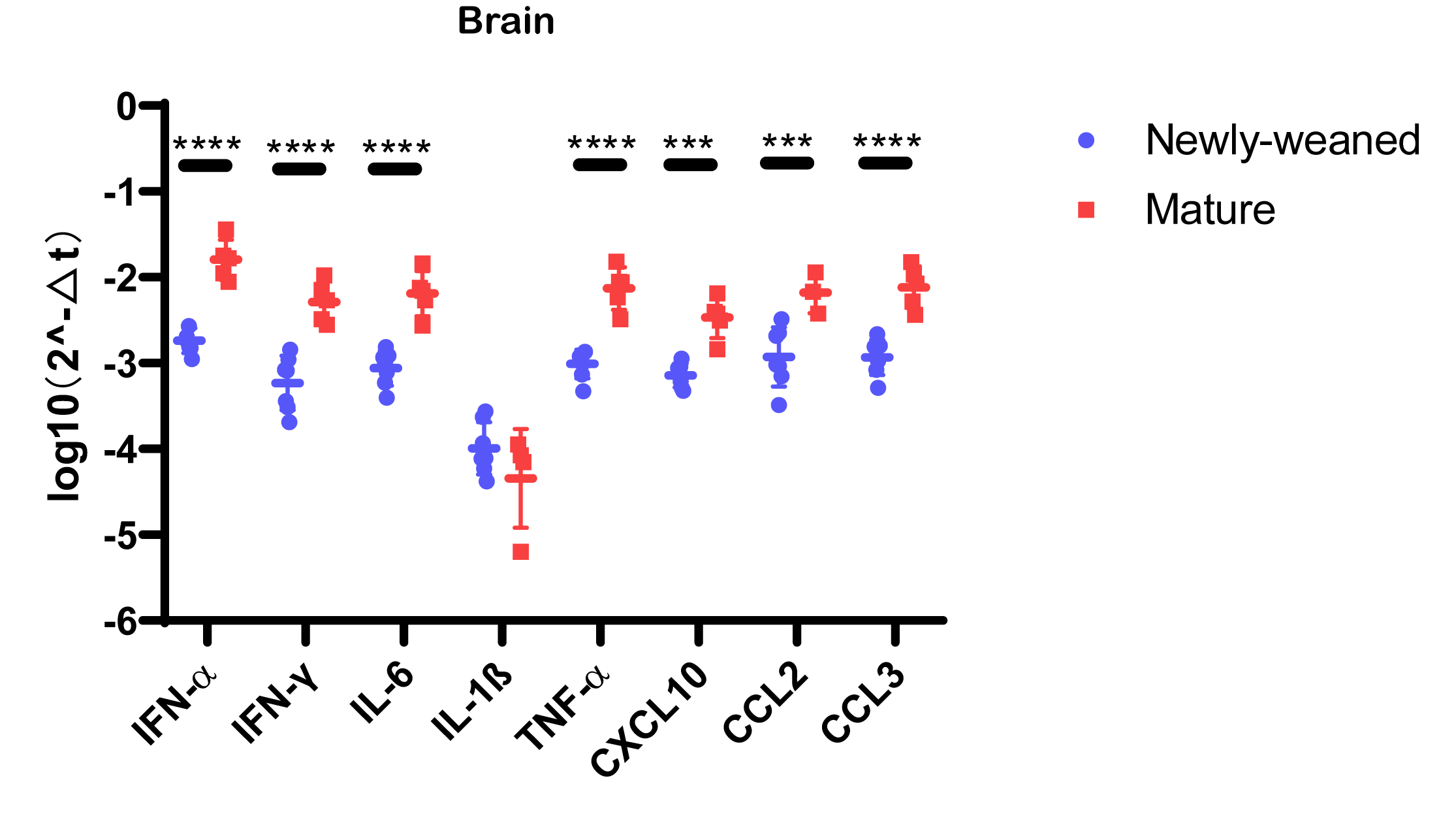

Supplement: Supplemental Material [file TEMI_A_2207678_SM2947.zip › Fig S2.tif]

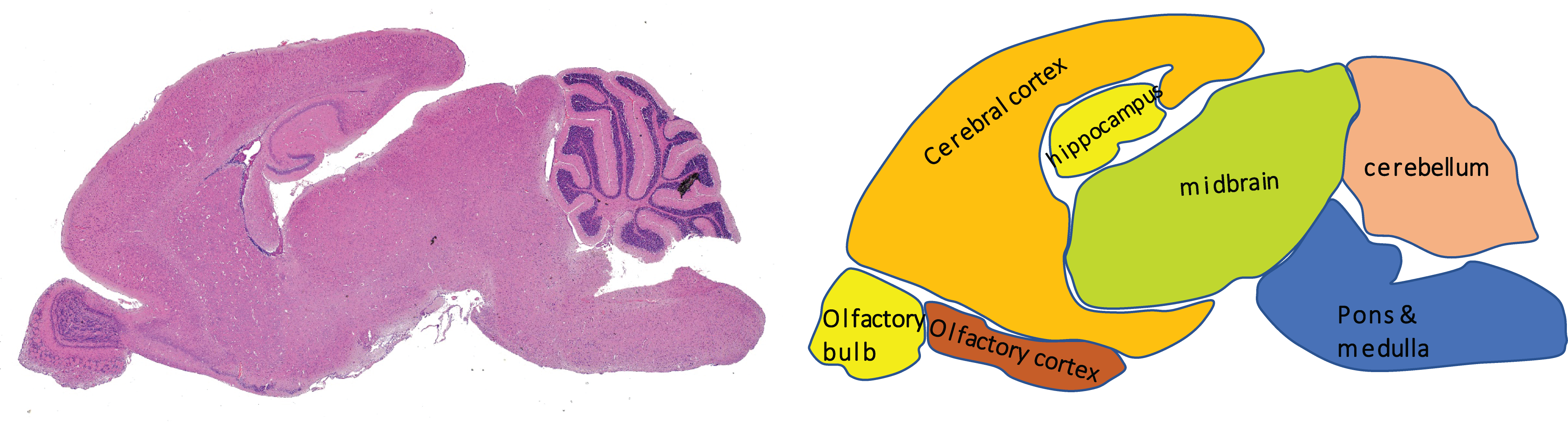

Supplement: Supplemental Material [file TEMI_A_2207678_SM2947.zip › Fig S3.tif]

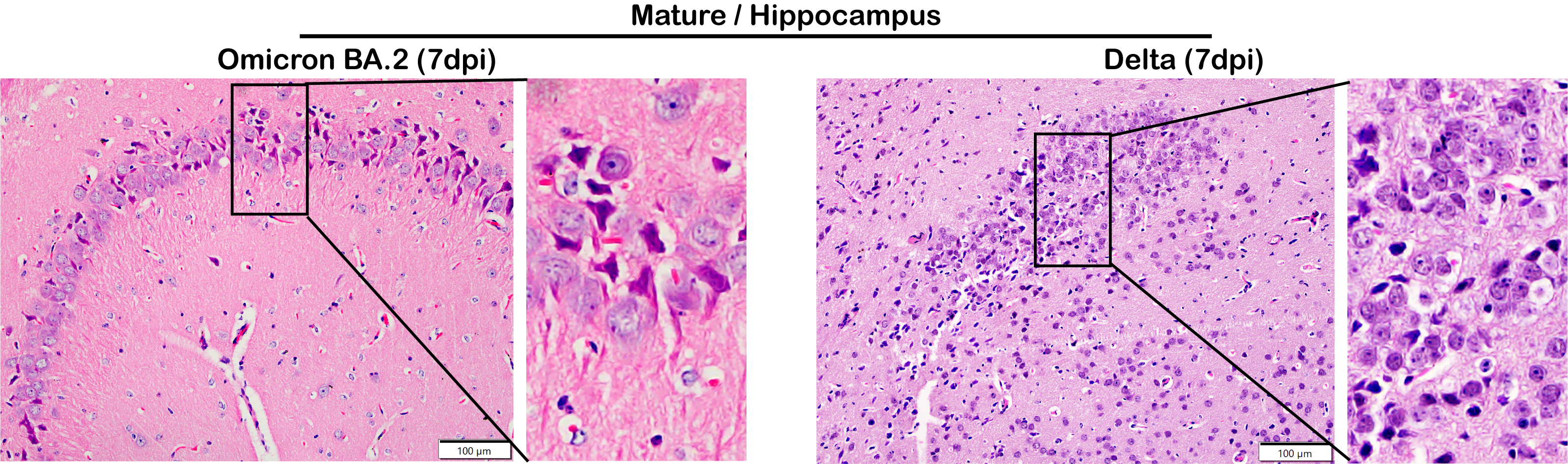

Supplement: Supplemental Material [file TEMI_A_2207678_SM2947.zip › Fig S4.tif]
